# Rabbit Facial Nerve Anastomosis with Fibrin glue: Nerve Conduction Velocity Evaluation

**DOI:** 10.1016/S1808-8694(15)31066-1

**Published:** 2015-10-22

**Authors:** Francisco Aurelio Lucchesi Sandrini, Cosme Gay-Escoda, Edwaldo Dourado Pereira-Júnior

**Affiliations:** 1 PhD Student in Bucco-Maxillofacial Surgery and Trauma - FOP/UPE.; 2 PhD in Bucco-Maxillofacial Surgery and Trauma, Adjunct Professor - School of Dentistry - University of Pernambuco - FOP/UPE. Mailing Address: Edwaldo Dourado Pereira Júnior - Av. Gal. Newton Cavalcanti 1650 Tabatinga Camaragibe PE 54753-220. Tel. (0xx81) 3458-2867; 3 PhD, Coordinator of the Master's Program in Bucco-Maxillofacial Surgery and Trauma - School of Dentistry - University of Barcelona.; 4 School of Dentistry - University of Pernambuco (FOP/UPE)

**Keywords:** fibrin tissue glue, nerve conduction, nerve regeneration

## Summary

Aim: The aim of this study was to evaluate the use of fibrin glue on nerve anastomosis, and study conduction velocity obtained by surface electrodes. **Methods:** In this experimental model we evaluated nerve conduction velocity differences in the preoperative and postoperative periods, for the left facial nerve of 12 rabbits. Then, we evaluated whether there were correlations between conduction velocity and the number of postoperative regenerated axons. The sectioned nerves were anastomosed with fibrin glue. The muscle action potentials were obtained from surface electrodes. The stimulation electrode was placed immediately before the ear pinna (facial nerve trunk) and the recording surface electrode was placed on the quadratus labii inferioris muscle. **Results:** The facial nerve normal conduction velocity mean value was of 36.53 m/sec. On the postoperative period, the mean conduction velocity was approximately 81% of the normal mean value. A significant correlation was not observed between the postoperative conduction velocity and the number of regenerated axons (p=0.146). **Conclusion:** The fibrin glue can be used on nerve anastomosis in this animal model and nerve.

## INTRODUCTION

Epineural and fascicular sutures are the most used microsurgical techniques for nerve repair in modern times. The epineural suture is the most common procedure, it is less invasive and, consequently, causes less nerve damage during the procedure. Fascicular suture^1^, ^2^, ^3^, ^4^, ^5^, ^6^, ^7^, ^8^, ^9^ is theoretically better because the nerve is sutured both internally and externally, thus allowing fascicular anastomosis. Notwithstanding, excessive handling may increase trauma, inflammation, and induce degenerative alterations to the nerve tissue.

The foreign body reaction caused by the suturing wire (nylon) to the nervous tissue is another possible problem faced by those who use conventional suturing material^10^, ^11^, ^12^. Proper material and instruments, coupled to the use of a surgical microscope may considerably enhance the nerve tissue repair procedure^13^. The possibility of carrying out a nerve anastomosis that would replace conventional suturing by biocompatible substances that do not cause organic reactions and other procedure-related problems would be ideal. Such possibility has been the goal pursued by many researchers^14^, ^15^, ^16^.

Fibrin glue is a plasma-derived biologic concentrate of topical use, of which mechanism of action is similar to the last stage of the physiological coagulation (fibrinogen formation). The clot constituted by the glue is a physiologic component found in tissue repair, and this makes it different from other types of glue such as the cyanoacrylates, which bear high fibrin formation and is very difficult to use in moist tissue^17^, ^18^, ^19^, ^20^, ^21^, ^22^, ^23^, ^24^. The fibrin glue Tissucol® (Immuno AG, Viena, Austria) commercially presents itself in two components, fibrinogen and the catalyst that, mixed by means of the Duploject® system (Immuno AG, Viena, Austria), solidify and form a plasmatic clog. The fibrin glue causes early clot formation, avoids hematoma and accelerates the repair process^3^.

Small amounts of fibrin glue may be easily applied in order to perform nerve anastomosis. Fibrin glue does not cause foreign body reaction or scar tissue formation, and it also reduces nerve stump manipulation and avoids the use of nylon wires, that remain in close contact with the nerve tissue^2^,^16^,^25^, ^26^, ^27^, ^28^, ^29^, ^30^.

In order to check the results attained in the studies about reinnervation dynamics, we can do histologic and functional evaluations. The functional evaluation obtained through recording nerve conduction velocity through electromyography with surface electrodes may be useful to stimulate and record muscle action potential and evaluate facial nerve functional status after an injury or surgery^31^.

The present investigation aims at using nerve conduction velocity with surface electrodes to assess fibrin glue as an alternative to conventional nerve anastomosis methods.

## MATERIALS AND METHODS

For this study, we used 12 white New Zealand male rabbits weighing between 2.5 and 3.0 Kg. The sample number was defined in a non-probabilistic fashion, following the principles advocated by the Spanish Standards of Animal Experimentation. This division in subgroups was carried out according to the periods defined for the assessment of nervous conduction velocity. All the animals were kept under ideal environmental conditions (25°C, 10% to 55% of relative air moisture). In order to carry out the surgical procedure and obtain the conduction velocities, the animals were put under general anesthesia with ketamine chloridrate 50mg/kg (Ketolar®; ParkeDavis, El Prat de Llobregat, Barcelona, Spain) and acepromazine maleate 5 mg/kg (Calmo Neosan®; SmithKlineBeecham, Madrid, Spain). Before the anesthesia induction, subcutaneous atropine was used (Atropina; Palex, Barcelona, Spain) for its antispasmodic and anticholinergic effects. After disinfection of the surgical field with iodine-povidine (Betadine; Asta Médica, Madrid, Spain), general anesthesia was complemented with local anesthesia by the administration of 3% mepivacaine with epinephrine 1:100.000 (Inibsa, Barcelona, Spain) by subcutaneous injection.

### Surgery and nerve injury

**Figure 1:**
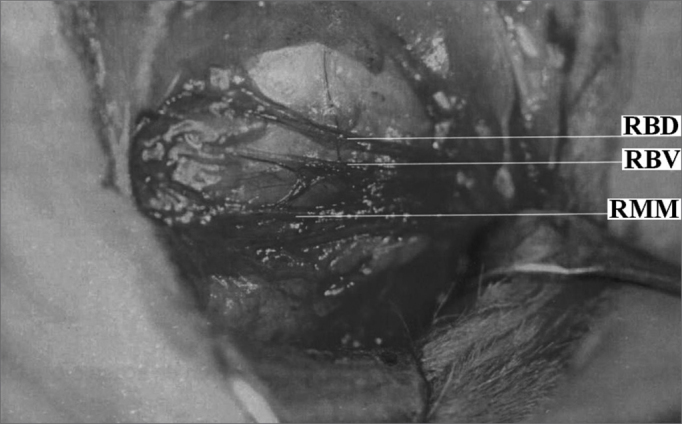
Facial nerve surgical anatomy. RBD Dorsal buccal branch; RBV Ventral buccal branch; RMM Mandibular marginal branch

**Figure 2:**
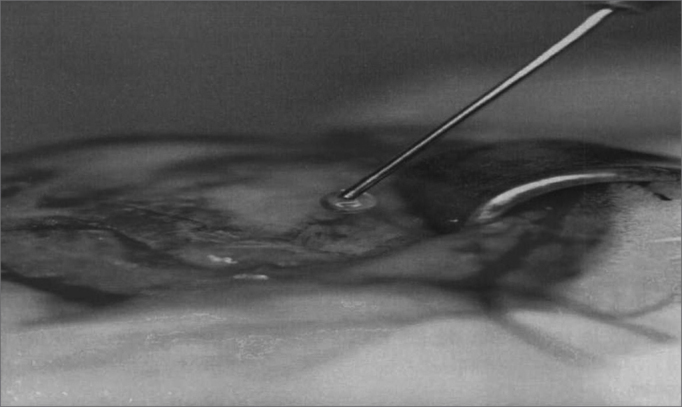
Fibrin glue use.

**Figure 3:**
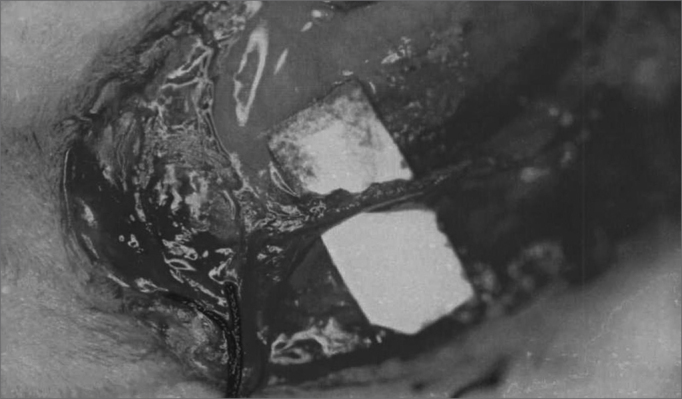
Nerve anastomosis with fibrin glue.

The anesthetized animal was then placed in lateral decubitus and we proceeded with pre-auricular and left neck hair trimming. We then, carried out a submandibular incision large enough to expose the facial nerve branches: dorsal buccal, ventral buccal and marginal mandibular (Figure 1). After exposing the facial nerve branches, the local anesthetic was then dripped directly on them in order to avoid the excessive use of general anesthesia. The dorsal buccal branch was then sectioned with the Barraquer microsurgery scissors (Hu-Friedy, Chicago, EUA) and joined with fibrin glue (Tissucol®, Immuno AG, Viena, Austria) (Figures 2 and 3). In all the animals we made a proximal and distal mark to the nerve anastomosis using a 3-0 silk suture thread (BraunDexon, Barcelona, Spain) in order to prevent sample removal for histology purposes from any region outside the operated field. The skin was then sutured with 3-0 silk suture thread (BraunDexon, Barcelona, Spain). Pre-operative analgesia was carried out with metamizol 30 mg/kg of body weight (Nolotil®, Europharma, Barcelona, Spain). All sutures were removed 15 days after surgery.

### Histologic and electrophysiologic analysis

Under general anesthesia, nerve conduction velocity was determined through surface electrodes. The stimulation electrode was placed immediately in front of the pinna (facial nerve trunk) and the recording electrode was placed on the quadratus labii inferioris. The evoked muscle action potential was recorded by a programmable 5 channel electromyograph ((Medelec MS25, Mistro, Surrey, U.K). Nerve conduction velocity was measured in meters per second and obtained in the pre and post operative periods (immediately before nerve harvesting for hystology purposes). The 12 animals were divided in 4 groups of equal size and slaughtered by a cardiac injection of sodium pentobarbital in overdose at 15, 30, 60 and 120 days after nerve anastomosis. We then calculated the conduction velocity difference (reduction) between the preoperative test and the postmortem test (postop). The surgical procedure and the functional evaluation (nerve conduction velocity) were carried out only on the left side, since there are no statistically significant differences between nerve conduction velocity of the right and left facial nerves in rabbits^31^.

For histology purposes we obtained cross sections from the distal portion of the facial nerve (near the anastomosis region). The surgical specimens were fixed in 10% glutaraldehyde solution and dyed with toluidine blue. We also carried out cross sections of the buccal dorsal branch and examined those under light microscopy at 400X magnification power. Axon count average was calculated by the average count of 4 randomized fields from each nerve.

Postop nerve conduction velocity and the number of regenerated fibers were then correlated.

### Statistical Analysis

Data analysis was carried out using the SPSS 11.0 (Statistical Package for Social Sciences, Illinois, Chicago, EUA). Conduction velocity pre and post surgery were compared by using the Wilcoxon test for paired data. The correlation between conduction velocity and regenerated axons count was evaluated by the Pearson's correlation analysis.

## RESULTS

Table 1 summarizes the results from this study.

**Table 1:** Facial Nerve conduction speed and axons count in animals submitted to fibrin glue anastomosis.

Follow up duration (days)	Nerve Conduction Speed (m/sec)*
Preoperative	36,53 ± 4,40**
15	20,13 ± 12,56
30	25,57 ± 6,41
60	23,67 ± 4,56
120	29,63 ± 2,76
Postoperative (Total)	24,76 ± 7,36

The other values were obtained in subgroups of 3 animals each.

Source: Research Data

* Values expressed in Mean Value ± Standard Deviation;

** This preoperative conduction velocity was calculated in 12 rabbits.

### Nerve conduction velocity

**Chart 1:**
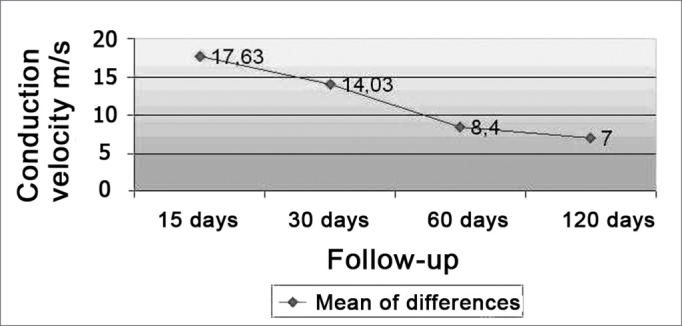
Differences of conduction velocity mean values between the pre and post operative periods by subgroups.

Source: Research Data

Nerve conduction velocity was measured in the 12 animals studied. Pre-operative conduction velocity mean value was of 36.53 ± m/sec. Maximum conduction velocity was of 46.10 m/sec. For postop follow up purposes, the animals were divided in 4 groups of 3 animals each (according ding to the nerve conduction speed evaluation periods). At 15 days, it was of 12.56 m/sec, conduction velocity mean value was of 20.13 ± 11.86 m/sec. Pre and postoperative mean values difference was of 17.63 ± 30 days after surgery, conduction mean velocity was of 25.57 ± 6.41 m/sec, pre and postoperative mean value difference was of 14.03 ± 12.00 m/sec. At 60 days of assessment, mean conduction velocity was of 23.67 ± 4.56 m/sec., the difference between pre and postoperative values was of 2.66 m/sec. 120 days after surgery, mean velocity was of 8.40 ± 2.76 m/sec., the difference between the pre and conduction values was of 29.63 ± postoperative were of 7.00 ± 1.25 m/sec. There were no significant differences between pre and postoperative values among the groups(Wilcoxon test: p= 0.109).Chart 1 depicts the mean conduction velocities differences between pre and postoperative periods broken down by subgroups. At 7.36 global postop mean velocity of all the animals was of 24.76 ± m/sec. The total mean value of conduction velocity values pre and postop was of 11.77 ± 8.57 m/sec. There was a significant difference when all conduction velocities were compared in the preoperative and all post op conduction velocities (Wilcoxon test: p = 0.002).

### Postoperative axonal regeneration

**Figure 4:**
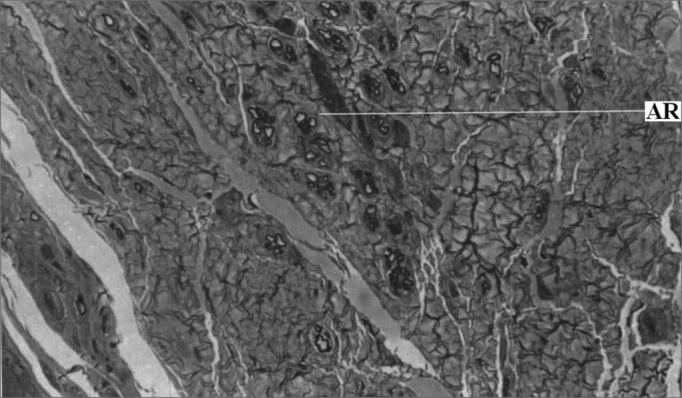
Left Facial Nerve Micro-photography after fibrin glue anastomosis (Toluidine Blue; 400x magnification).

Legend: AR Regenerated axons.

After 15 days of nerve anastomosis with fibrin glue, there was no sign of axonal regeneration. From 30 to 120 days of postop, there was an increase in axon count with time. There was no significant correlation between postop conduction velocity and axonal regeneration (Pearson's test: p=0.146). Figure 4 depicts the nerve specimens used for axon count.

## DISCUSSION

Fibrin glue induces nerve stump anastomosis, reduces nerve joining difficulty, and reduces damage to peripheral nerve at the joining point. Fibrin glue is permeable and remains in place for the necessary time to allow nerve stump union^32^,^33^.

Young and Medawar^34^ introduced this material as an alternative to conventional nerve suturing. These authors reported that sectioned nerves could be joined by means of a plasma-derived concentrated substance that coagulates around the nerve. In this study, the glue was not enough to keep the stumps together under stress. In this context, both adhesiveness and retraction related to the use of this glue have remained as problems for decades now^35^, ^36^, ^37^. This problem was solved later on with the increase in fibrinogen concentration in this type of glue.

Fibrinogen's fundamental reaction is its conversion into fibrin. Fibrinogen is soluble in plasma and its conversion is catalyzed by thrombin, which is formed by prothrombine in response to the activated factor X action and calcium ions. In order to reach the thrombin required concentration, a calcium chloride solution is applied to the dried-frozen thrombin at a 500 UI/L concentration (for slow clotting)^38^,^39^.

The fibrin glue components mixture used in this study is carried out by means of a system with two syringes (Duploject®, Immuno AG, Viena, Austria), which blend the components in the application needle, thus avoiding clotting before product use. After application, the needle may be replaced, thus allowing multiple applications with the same glue unit within a maximum period of 4 hours.

Fibrin glue reactions are similar to those seen in regular physiological clotting: no significant clot retraction is seen40. Fibrin glue may be used as an alternative to conventional suturing in different situations, in the maxillofacial region, and its use in septoplasties prevents hematoma and hemorrhage in the postop period^41^.

As to nerve repair, its application is safe and histologic and electrophysiologic results are favorable. Nonetheless, results related to nerve tension and suture retraction caused by conventional suturing are better^32^. The fibrin glue used in the present study (Tissucol®, Immuno AG, Viena, Austria) was the same one used by Maragh et al.^32^.

Many authors^5^, ^6^, ^7^,^16^,^18^,^22^,^30^,^34^,^42^,^43^ have analyzed new anastomosis alternatives in order to optimize nerve regeneration. The main options include the use of artificial nerve conduction means, CO_2_ laser and tissue adhesive or glue.

Tissue adhesive may be synthetic or biologic. Among existing adhesive we may stress the cyanoacrylates. However, they induce fibrosis and foreign body reaction, thus not being recommended for nerve anastomosis^33^.

In order to assess nerve anastomosis with the fibrin glue in the present study we carried out functional and histology analyses. After 15 days of postop, no regenerated axons were seen in histology. From 30 to 120 days of postop, there was a growing increase in axon count, despite the low number of regenerated axons.

In order o assess facial nerve functional status, electromyography proved very useful, because it is able to stimulate and record muscle action potential. Surface electrodes are able to record the nerve's functional status after the trauma or surgery, in a non-invasive fashion^31^.

In the present study, nerve conduction velocity was determined in all the animals by means of surface skin electrodes, avoiding damage to nerve and muscle^5^, ^6^, ^7^. Average facial nerve conduction velocity in rabbits is of 42.29 m/sec according to Gay-Escoda et al.^6^; and of 41.1 m/sec according to Vasconcelos7. In the present investigation, the velocity was of 4.40 m/sec. All these results are within normal standards for the nerve and the animal in question.

In the present study, nerve conduction velocities obtained in the 15 day postop period represented 53.4% of the preoperative (initial) value. In the 30 day postop period, the values found were 64.52% of the initial value. In the 60 day postop period, values represented 73.8% of the pre-operative value. In the 120th day of postop, values were 80.9% of the initial value. In the global period, postop values represented 67.8% of the normal value.

Nerve conduction velocity may be obtained through the use of surface electrodes, internal electrodes and needle electrodes. The use of surface electrodes allows for a very precise and non-invasive functional evaluation, thus avoiding another surgical procedure (required by the internal electrodes). It also precludes the possibility of muscle or nerve injury and infection at the implantation site, which may occur when one uses needle electrodes^31^,^44^,^45^.

Despite the low number of regenerated axons, nerve conduction speed showed a good facial nerve recovery with fibrin glue, reaching a value close to 81% of the normal value at the end of the period. This functional data certifies fibrin glue to be used as an alternative for nerve joining in this model of animal injury. Nerve anastomosis with fibrin glue is technically simple and apparently carried out in less time than epineural suturing (although this piece of data was not measured in this study).

It is necessary to carry out studies comparing epineural suturing with fibrin glue in human nerve injuries, in order to guarantee that the fibrin glue presents results that are similar to conventional suturing.

## CONCLUSION

Fibrin glue may be used in order to carry out nerve anastomosis in the animal and nerve models we studied.
